# Hair shaft structures in *EDAR* induced ectodermal dysplasia

**DOI:** 10.1186/s12881-015-0227-5

**Published:** 2015-09-04

**Authors:** C. Stecksén-Blicks, C. Falk Kieri, D. Hägg, M. Schmitt-Egenolf

**Affiliations:** Pediatric Dentistry, Department of Odontology, Faculty of Medicine, Umeå University, Umeå, Sweden; Dermatology, Medicine, Department of Public Health and Clinical Medicine, Faculty of Medicine, Umeå University, Umeå, Sweden

## Abstract

**Background:**

Mutations in the *EDAR*-gene cause hypohidrotic ectodermal dysplasia with defects in ectodermal appendage development including teeth, skin, exocrine glands and hair. Hair defects are sparsely described in genetically defined samples. The aim of this study was to investigate hair structures in three families with a heterozygous c.1072C > T mutation in the *EDAR* gene using scanning electron microscopy.

**Methods:**

Three Swedish families, where some members had a known c.1072C > T mutation in the *EDAR* gene with an autosomal dominant inheritance (AD) were included (*n* = 37) of which 17 carried the mutation and 20 did not. Thirty-two age and gender matched not related individuals served as a reference group. Confirmation of the c.1072C > T mutation in the *EDAR* gene was performed by genomic sequencing. Hairs were subjected to blinded scanning electron microscopy examination and hair defects were categorized and scored.

**Results:**

The minimum and maximum diameters of hairs were lower in the mutation group compared to the reference group. Subjects in the mutation group had to greater extent deep deformations in hair shafts compared to the non-mutation group and the reference group (p < 0.001).

**Conclusions:**

Individuals with a c.1072C > T mutation in the *EDAR-*gene displayed more hair shaft deformations confirming the role of *EDAR* for human hair follicle development and postnatal hair follicle cycling.

## Background

Ectodermal dysplasias (EDs) are genetic disorders with lack or dysgenesis of at least two of the ectodermal derivatives; hair, nails, teeth or sweat glands [1]. Yavuz et al [2] reported that the most frequent abnormality in ectodermal dysplasia is skin disorders (93 %), followed by hair and nail disorders (86 %). Hypohidrotic ectodermal dysplasia (HED) which is the most common form of ectodermal dysplasia is characterized by severe defects in ectodermal appendage development, including hairs, teeth, and exocrine glands. Common symptoms in individuals with HED are reduced number of teeth, reduced saliva secretion, dry skin and sparse and thin hair [3]. HED can be inherited in X-linked, autosomal dominant (AD) or autosomal recessive (AR) manner. Four genes (*EDA1, EDAR, EDARADD and WNT10A)* account for 90 % of hypohidrotic ectodermal dysplasia cases [4] and mutation in *EDAR* have been reported to account for 25 % of non-*EDA1* HED cases [5].

Hair shaft phenotypes in ectodermal dysplasia have been reported from mainly genetically undefined samples. In a three generation family with putative AD HED, scanning electron microscopy showed longitudinal grooves in the hair shafts and defective cuticular layers [6]. In another study structural hair abnormalities were found in a group of patients with phenotypically heterogeneous ED [7]. Furthermore, structural abnormalities such as twisted hairs and longitudinal grooves were found in hair samples from patients with hypomelanosis of Ito and some other ectodermal dysplasias [8]. In X-linked hypohidrotic ectodermal dysplasia with an *EDA1* mutation fewer hair follicles and hairs with decreased thickness were observed [9].

The hair follicle is a skin appendage that develops in epithelial-mesenchymal interactions between epidermal keratinocytes committed to hair-specific differentiation and a cluster of dermal fibroblasts that forms the follicular papilla. Mice studies documented the role of Edar signalling for i) the control of hair follicle development and growth that takes place in a sequential series of epithelial–mesenchymal interactions, as well as ii) postnatal hair follicle regeneration by regulating the apoptosis in keratinocytes. This takes place in a cross-talk between Edar and other signalling pathways. The Wnt pathway is considered to be the master regulator during hair follicle morphogenesis and Wnt signalling proceeds through *EDA/EDAR/NF-kB* signaling. *NF-kB* regulates the Wnt pathway and acts as a signal mediator by upregulating the expression of Shh ligand [10–15]. Effects on human hair structure related to deficient *EDAR* signaling have previously not been described.

Previously we characterized individuals with a heterozygous c.1072C > T mutation (p.ARG358X) in the *EDAR* gene with respect to dental signs and symptoms from other ectodermal structures but not hair [16]. The aim of the present study was to investigate hair structures in three families with a heterozygous c.1072C > T mutation in the *EDAR* gene using scanning electron microscope. The null hypothesis was that there were no differences compared to family members without the mutation.

## Methods

Forty-six members of three families, where some had a known mutation in the *EDAR* gene [17], were invited to participate in a clinical examination of signs and symptoms from ectodermal structures. Nine family members declined participation and thus the material consisted of 37 subjects. Confirmation of the c.1072C > T mutation in the *EDAR* gene was performed by genomic sequencing [16]. Seventeen out of the 37 family members carried the mutation in the *EDAR* gene. The reference group consisted of 32 individuals who were age and gender matched to all subjects with the mutation, and to 15 of 20 individuals without the mutation. The mean age of the family members was 31.5 ± 23.1 years; 35.6 ± 22.6 in the mutation group and 28.2 ± 23.7 in the non-mutation group (p > 0.05); 49 % were males, 47 % in the mutation group and 50 % in the non-mutation group (p > 0.05). One male individual with the known mutation was completely bald and consequently not included in the hair analysis.

Written informed consent was received from all participating individuals. Parents provided consent for individuals below 18 years of age. The study was approved by The Regional Ethical Review Board in Umeå, Sweden (Dnr 2011-123-32 M).

Five to 15 hairs were collected from each participant and they were stored dry at room temperature in a plastic bag until examination of scale structure of cuticle layer, diameter, deformation-grade, and deformation-extent using scanning electron microscopy. All hairs were in its native state, and not chemically treated prior to the examination. All participants regularly used shampoos for cleaning and some had used additional products such as conditioners, wax and gel. Before examination the purity of the hairs were assessed. The scanning electron microscopy examinations were performed blinded at the electron microscope platform at Umeå University by an experienced examiner and the maximum and minimum diameters of hairs were measured as well as variation of diameters. Still blinded, scale structure of cuticle layer and deformations were categorized from photos and scored according to the criteria in Table 1.

**Table 1:** Scoring of variables assessed in electronic microscopy

Variables	Scores/measure
Purity	1. Completely clean
	2. Relatively clean
	3. Not completely clean
Scale structure of cuticle layer	1. Normal
	2. Incipient loss of structure
	3. Almost no structure
	4. Smooth surface
	5. Erosions
	6. Reinforced scale structure
Diameter min	μm
Diameter max	μm
Variation in diameter	1. No variation
	2. Continuous variation of the same size
	3. Large local variation
Deformation grade	1. None
	2. Superficial (no obvious grooves present)
	3. Deep (obvious grooves present)
Deformation extent	1. None
	2. Patchy
	3. Large surface areas
Twisted hair	1. No
	2. Yes

### Statistical analysis

The results for the categorical variables are presented in percentages for the three groups, while the continuous variables are described by mean, standard deviation (sd), median and interquartile range (IQR). The difference between the groups was tested by Fisher exact test for the categorical variables and by the Mann–Whitney–Wilcoxon or Kruskal-Wallis rank sum test for the interval scale measurements. A p-value of < 0.05 was considered as statistically significant.

## Results

### Scale structure and diameters of hair

**Fig. 1:**
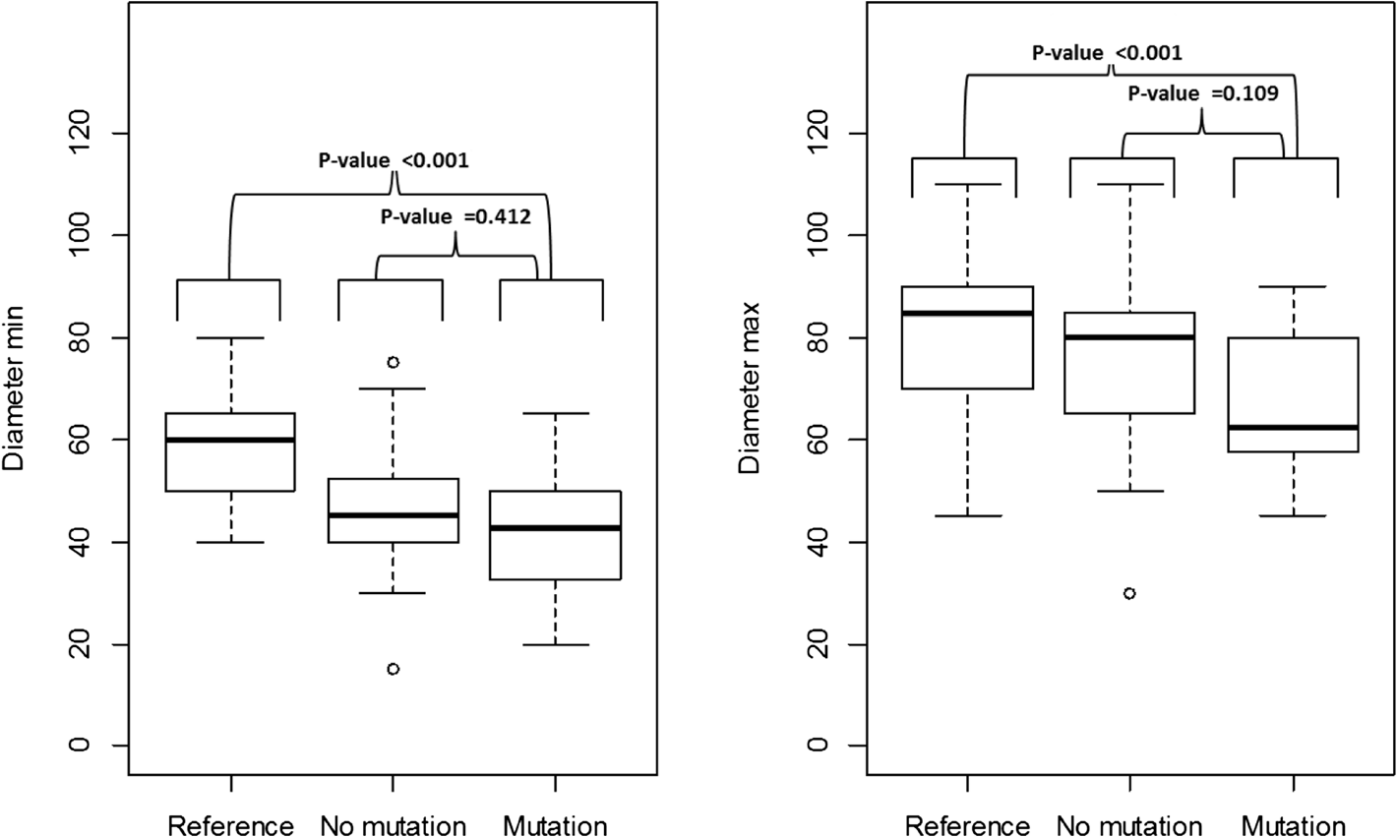
Minimum and maximum diameters of hair. Boxes show 50 % of the cases, lines across boxes denote median values, and whiskers show the IQR × 1.5 ± Q1/Q3 and outliers are indicated with a circle

No noticeable differences were identified between the three groups regarding the scale structures of the cuticle layer of hair. The minimum and maximum diameters of hair were lower in the mutation group compared to the reference group (p < 0.001), (Table 2, Fig. 1). No statistically significant difference in minimum diameter (*p* = 0.422) or maximum diameter (*p* = 0.122) was found between the mutation group and non-mutation group. The variation of the hairs diameter did not differ between the mutation group and the non-mutation group (*p* = 0.448) or between the mutation group and the reference group (*p* = 0.254).

**Table 2:** Distribution of gender, number of examined hairs, scored scale structure and deformation variables

	Reference group	Non-mutation group	Mutation group
	*n* = 32	*n* = 20	*n* = 17^a^
Women, n (%)	17 (53.1 %)	10 (50.0 %)	9 (56.3 %)
Examined hairs, mean (sd)	9.5 (2.2)	10.2 (2.0)	9.6 (2.3)
Scale structure of cuticle layer			
1. Normal	23	12	8
2. Incipient loss of structure	2	1	1
3. Almost no structure	3	2	2
4. Smooth surface	3	5	1
5. Erosions	1	0	3
6. Reinforced scale structure	0	0	1
Diameter (Min/Max) μm	40/110	15/110	20/90
Variation in diameter μm			
1. No variation	0	0	1
2. Continuous variation of the same size	31	17	14
3. Large local variation	1	3	1
Deformation grade			
1. None	7	2	0
2. Superficial (no obvious grooves present)	24	15	4
3. Deep (obvious grooves present)	1	3	12
Deformation extent			
1. None	7	2	0
2. Patchy	8	13	6
3. Large surface areas	17	5	10
Twisted hair	0	0	2
Baldness	0	0	1

^a^One subject bald and consequently not included in hair analysis

### Deformation of hair

**Fig. 2:**
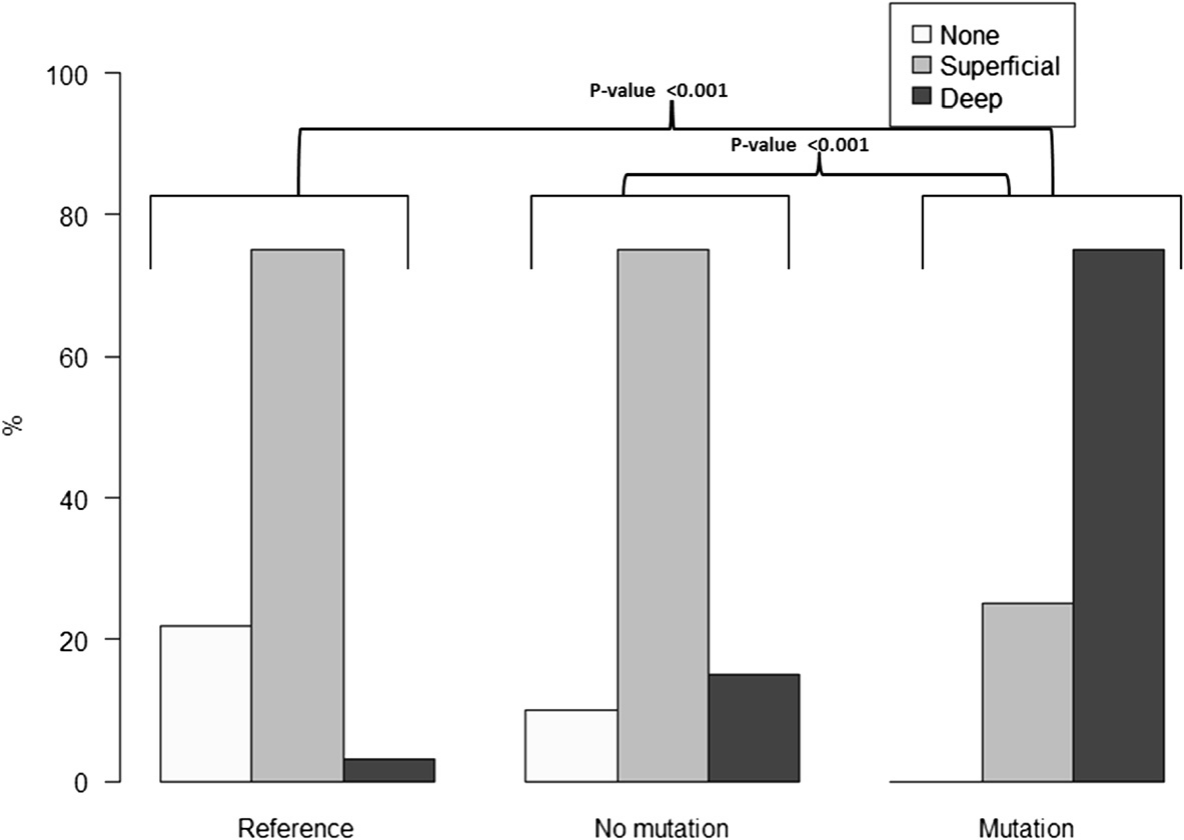
Deformation grade of hair in the reference group (*n* = 32), non mutation group (*n* = 20) and mutation group (*n* = 16)

**Fig. 3:**
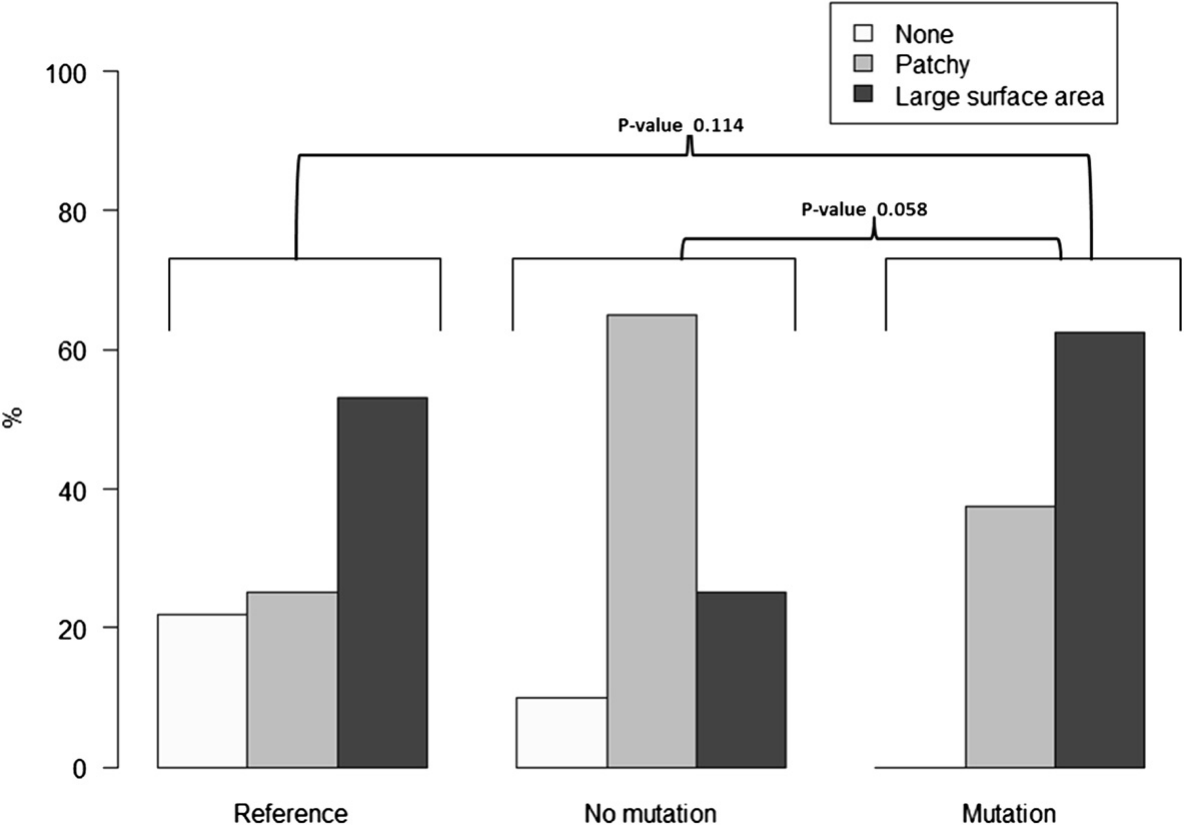
Deformation extent of hair in the reference group (*n* = 32), non mutation group (*n* = 20) and mutation group (*n* = 16)

**Fig. 4:**
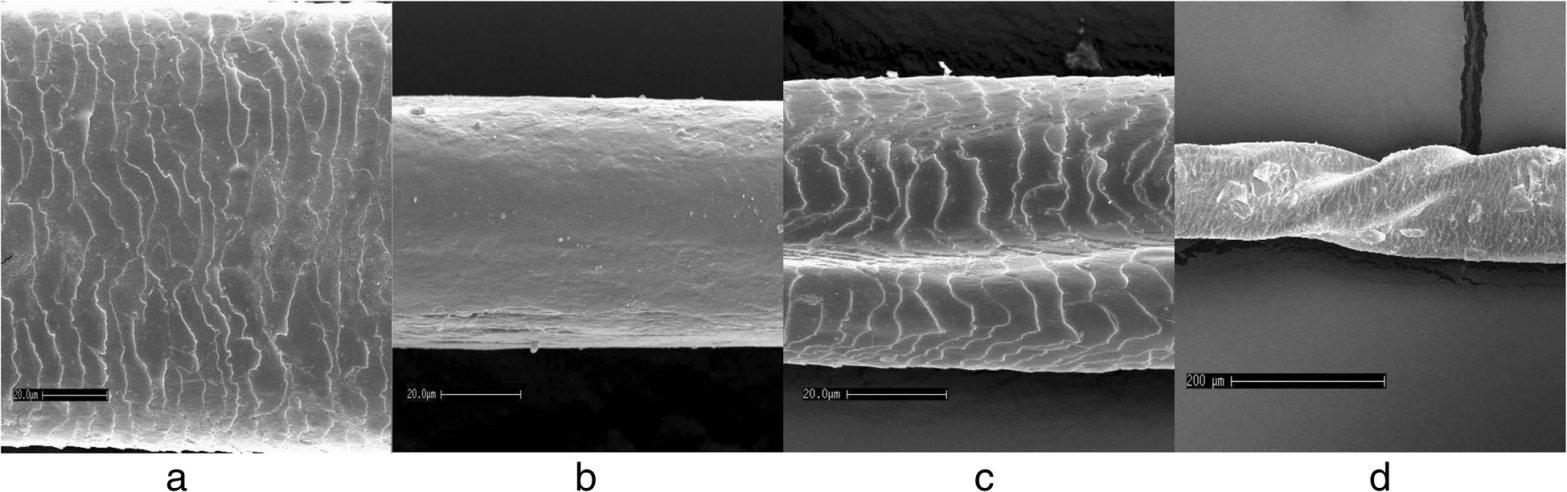
Examples of hairs. **a** Normal scale structure and no abnormalities. **b** Lack of scale structure. **c** Deformation grade deep. **d** Twisted hair

The proportion of individuals with a deep deformation grade was highest in the mutation group (75.0 %) compared to the non-mutation group (15.0 %) and reference group (3.1 %), (Table 2). The difference was statistically significant between the mutation group compared to the non-mutation group and compared to the reference group, (p < 0.001) for both comparisons while no statistically significant difference could be identified between the non-mutation group and the reference group (p > 0.05), (Fig. 2). Sixty-two per cent in the mutation group, 25 % in the non-mutation group 53 % in the reference group had deformations on large surface areas of the hair (Table 2), but the differences were not statistically significant between the mutation group and the non-mutation group (*p* = 0.058) or between group of the mutation and the reference group (*p* = 0.114), (Table 2, Fig. 3). Two male participants in the mutation group had twisted hair compared to none in the non-mutation and none in the reference group (Fig. 4).

## Discussion

This study describes the effect on hair structure of a heterozygous c.1072C > T mutation (p.ARG358X) in the *EDAR* gene which has previously not been described in a genetically defined human clinical material. Deep hair deformations were more common in the mutation group and the null-hypothesis could therefore be rejected for deformation of hairs. The mutation which changes a CGA arginine codon to a TGA stop codon has earlier been described [17].

Structural hair defects may be a marker for an underlying metabolic disorder, the expression of a genetic disorder affecting hair growth or part of a congenital syndrome with accompanying hair malformations [18]. The subjects in the mutation group fulfilled the criteria of having a congenital syndrome because of the combination of missing teeth due to agenesis, aberrant tooth form, reduced salivary secretion and reduced sweating ability [16]. Therefore, the structural hair defects can be seen as an additional developmental defect linked to the dysfunctional *EDAR* gene. Our characterisation of hair phenotype of the heterozygous c.1072C > T mutation in the *EDAR* gene gives a clinical confirmation for the role of *EDAR* for human hair follicle development and postnatal hair follicle cycling. The corresponding role of Edar in mice has earlier been shown [12]. The comparison with both the related non-mutation group and the reference group is a strength of this study. To control for a familial impact on hair deformations and thickness we selected an age- and gender matched not related reference-group.

There was a clear difference of hair diameters, both in minimum and maximum diameters, between the mutation group and the non-related reference group. However, we detected no difference between the mutation group and the related non-mutation group. Our data may therefore reflect a familial impact on hair thickness but not on hair deformations. This indicates that there are other genetic variants associated with hair thickness [19]. Fewer hair follicles and lower average terminal hair diameter were seen in 12 males with X-linked ectodermal dysplasia with an *EDA1* mutation compared to 13 non-related controls. However, no comparison with related healthy family members was performed [9]. The average reported thickness of hairs was 51 μm for males with the mutation and 71 μm for the control group and these figures correspond well with our data. We did not assess number of hair follicles. However, it was a clinical impression that many subjects in the mutation group had sparse hair and consequently fewer hair shafts than family members that did not carry the mutation. Sparse hair were described clinically in a large family with AD HED without any comparison with un-affected family members [20]. Interestingly, the deformations in hair shafts shown in the present material resembles the longitudinal grooves in hairs described by Jorgensen et al. in a three generation family with putative AD HED [6] and by Selvaag et al. in a group with different ED’s [8].

We have earlier shown that the deficiency in *EDAR* signaling because of a heterozygous c.1072C > T mutation also is associated with a higher number of missing teeth due to agenesis, reduced saliva secretion and reduced ability to sweat [16]. However, the severity of sign and symptoms were generally milder than in the more common *EDA1* mutations, particularly for sweating problems [9, 21] and varied between individuals. Variations between individuals who have the mutation were also obvious in hair deformities. The scanning electron microscopic examination showed thin twisted hair in two male subjects in the mutation group; they were both affected with severe problems of dry skin and had severe oligodontia. The 22-year-old brother to one of the subjects with twisted hair in the mutation group was suffering from complete baldness since before the age of 20 and could consequently not be included in the hair analysis. He was congenitally missing 14 permanent teeth except third molars. Unfortunately, although this individual had baldness as the most severe expression of a hair mutation, he could not be in included in the statistical analyses of the hair anomalies and increase the statistical power.

## Conclusion

Individuals with a heterozygous c.1072C > T mutation in the *EDAR-*gene displayed more hair shaft deformations confirming the role of *EDAR* for human hair follicle development and postnatal hair follicle cycling.
